# Review on electrical stimulation combined with electroactive biomaterials to promote peripheral nerve regeneration

**DOI:** 10.1093/burnst/tkaf039

**Published:** 2025-05-31

**Authors:** Jiahui Song, Zhengchao Yuan, Xiao Yu, Yihong Shen, Jinglei Wu, Binbin Sun, Cheng Xue Qin, Mohamed EL-Newehy, Xiumei Mo, Hongbing Gu

**Affiliations:** School of Food and Pharmacy, Shanghai Zhongqiao Vocational and Technical University, No. 888 Caolang Road, Jinshan District, Shanghai 201514, P.R. China; State Key Laboratory for Modification of Chemical Fibers and Polymer Materials, Shanghai Engineering Research Center of Nano-Biomaterials and Regenerative Medicine, College of Biological Science and Medical Engineering, Donghua University, No. 2999 North Renmin Road, Songjiang District, Shanghai 201620, P.R. China; State Key Laboratory for Modification of Chemical Fibers and Polymer Materials, Shanghai Engineering Research Center of Nano-Biomaterials and Regenerative Medicine, College of Biological Science and Medical Engineering, Donghua University, No. 2999 North Renmin Road, Songjiang District, Shanghai 201620, P.R. China; State Key Laboratory for Modification of Chemical Fibers and Polymer Materials, Shanghai Engineering Research Center of Nano-Biomaterials and Regenerative Medicine, College of Biological Science and Medical Engineering, Donghua University, No. 2999 North Renmin Road, Songjiang District, Shanghai 201620, P.R. China; State Key Laboratory for Modification of Chemical Fibers and Polymer Materials, Shanghai Engineering Research Center of Nano-Biomaterials and Regenerative Medicine, College of Biological Science and Medical Engineering, Donghua University, No. 2999 North Renmin Road, Songjiang District, Shanghai 201620, P.R. China; State Key Laboratory for Modification of Chemical Fibers and Polymer Materials, Shanghai Engineering Research Center of Nano-Biomaterials and Regenerative Medicine, College of Biological Science and Medical Engineering, Donghua University, No. 2999 North Renmin Road, Songjiang District, Shanghai 201620, P.R. China; State Key Laboratory for Modification of Chemical Fibers and Polymer Materials, Shanghai Engineering Research Center of Nano-Biomaterials and Regenerative Medicine, College of Biological Science and Medical Engineering, Donghua University, No. 2999 North Renmin Road, Songjiang District, Shanghai 201620, P.R. China; Drug Discovery Biology, Monash Institute of Pharmaceutical Sciences, Monash University, 381 Royal Parade, Parkville, VIC 3052, Australia; Department of Chemistry, College of Science, King Saud University, P.O. Box 2455, Riyadh 11451, Saudi Arabia; School of Food and Pharmacy, Shanghai Zhongqiao Vocational and Technical University, No. 888 Caolang Road, Jinshan District, Shanghai 201514, P.R. China; State Key Laboratory for Modification of Chemical Fibers and Polymer Materials, Shanghai Engineering Research Center of Nano-Biomaterials and Regenerative Medicine, College of Biological Science and Medical Engineering, Donghua University, No. 2999 North Renmin Road, Songjiang District, Shanghai 201620, P.R. China; Department of Cardiovascular Surgery, Shanghai General Hospital, Shanghai Jiaotong University School of Medicine, No. 650 Xinsongjiang Road, Songjiang District, Shanghai 201600, P.R. China

**Keywords:** Peripheral nerve injury, Electrical stimulation, Electroactive biomaterials, Peripheral nerve regeneration

## Abstract

Peripheral nerve injury results in sensory and motor dysfunction, which is an enormous economic burden for patients and society. Complete recovery of peripheral nerve function after injury is complicated. Utilizing the electrophysiological properties of natural nerves for neuronal regulation and axon regeneration has attracted considerable interest. Electroactive biomaterials induce an active state of electrical stimulation (ES) at the site of peripheral nerve injury when incorporated into nerve guidance channels. Numerous studies have demonstrated that combining ES with electroactive biomaterials can enhance peripheral nerve repair. This review summarizes the regulation of signal pathways by ES and the functions of various electroactive biomaterials, including metals, carbon-based materials, conductive polymers, and piezoelectric materials. Recent advances and research of ES combined with electroactive biomaterials in peripheral nerve repair are reviewed, which may help to come up with more effective strategies to restore neural function after PNI.

## Background

Peripheral nerves form extensive neural networks throughout the body that transmit electrical signals between neural centers and target organs [1]. Peripheral nerve injury (PNI) is a common and widespread clinical disease triggered by acute trauma, autoimmune diseases, local lesions, and infections [2]. When PNI occurs, the motor and sensory functions of the distal target organs are blocked due to the inability to transmit information. The growth rate of injured peripheral nerves is ~1 mm/day, so regeneration of nerve defects takes a long time [3]. Concurrently, the target organs of distal innervation gradually atrophy during nerve regeneration, resulting in impaired sensory and motor function. Therapeutic strategies for peripheral nerve injury include nerve ends suturing, fibrin glue injection, applying exogenous electrical stimulation (ES), autologous nerve grafting, and utilizing nerve guidance conduits [4]. However, sutures and fibrin glue are ineffective for long-distance nerve defects, while exogenous ES devices have limited application [5,6]. Although autologous nerve grafting is considered the “gold standard” for long-gap injuries, it faces challenges such as donor site shortage, numerous postoperative complications, and suboptimal recovery rates [7–9]. Consequently, these traditional treatments often fail to achieve satisfactory clinical outcomes. Nerve guidance conduits (NGCs) provide a physical scaffold to guide the growth of regenerating axons and serve as a delivery vehicle for bioactive molecules or cells to support nerve regeneration. Importantly, NGCs avoid the secondary injury and donor site morbidity associated with autografting. These advantages have positioned NGCs as a focal point of research, marking a transition from the traditional era of autograft-based peripheral nerve repair to one dominated by artificial implantation technologies [10]. Various commercial NGCs are available, including designed membrane products primarily to protect injured peripheral nerve ends, such as NeuraWrap, NeuroMend, and conduits designed directly to bridge nerve gaps, such as Neutrogena, Neurotube, and Neurolac [11]. However, these commercial NGCs exhibit limitations in mechanical properties and bioactivity, which are prerequisites for functional and sensory recovery. Therefore, it is crucial to explore more ideal biomaterials and bioinspired functionalities for NGCs.

As an adjuvant therapeutic strategy, ES combined with electroactive biomaterials has been widely used to promote peripheral nerve regeneration. Biological electricity is critical for the maintenance of peripheral nerve function, which can control neuronal survival and axon extension [12]. This process involves switching on and off associated receptors and channels on the cell membrane of neurons [13]. Therefore, the use of exogenous ES to modulate cell activity and promote nerve repair is a viable strategy [14]. Electroactive biomaterials exhibit a pronounced ES response and support cellular activity under both external and internal ES conditions, making them an optimal candidate for functional NGCs [15]. In addition, electroactive biomaterials can use their intrinsic electroactivity to modulate the electrophysiological microenvironment of natural peripheral nerves and accelerate the nerve regeneration process [16].

This review summarizes the effects and mechanisms of ES on peripheral nerve growth. The application and development of various conductive materials in neural conduits in recent years are systematically introduced, and future trends in the development of NGCs are proposed, based on the response of electroactive biomaterials to ES. Hopefully, it will provide more compelling insights into the latest advances in peripheral nerve regeneration under the synergistic action of ES and conductive materials.

## Review

### Peripheral nerve injury and repair

**Figure 1:**
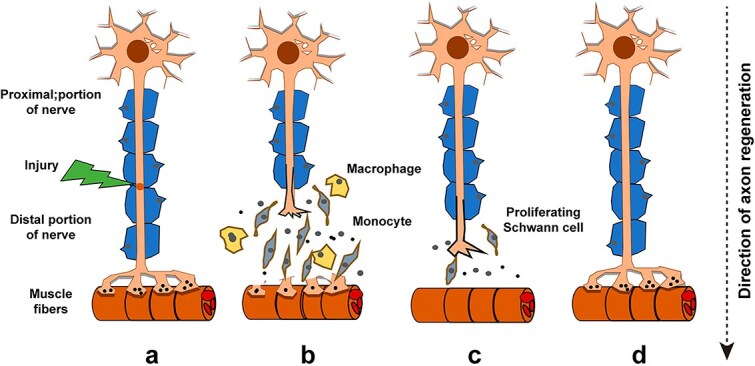
Wallerian or anterograde process after PNI. (**a**) Peripheral nerve transection, (**b**) immune cells clean up broken nerve fragments, (**c**) SCs proliferate to form Büngner bands, and (**d**) nerve regeneration. Reproduced with permission [9]. Copyright 2019, American Chemical Society

Peripheral nerve injury can be divided into several categories: neuropraxia, axonotmesis, neurotmesis, and nerve defect [17]. We will mainly focus on peripheral nerve injuries such as nerve defects. After peripheral nerve injury, disruption of axonal continuity leads to blockage of nerve signal transmission, ultimately resulting in partial or complete loss of motor, sensory, and autonomic functions [18]. After peripheral nerve injury, the immune system plays a leading role in coordinating the activities of Schwann cells (SCs), neurons, axons, fibroblasts, and myocytes *in vivo* to enhance the ability of nerves to recover [19]. During the repair process, peripheral nerves undergo a multi-step repair process of Wallerian degeneration, axon regeneration, and target nerve regeneration [20]. Among them, Wallerian degeneration is the deformation and disintegration of axons and myelin sheaths. Initially, SCs detached from damaged axons cooperate with macrophages streaming in from the blood–nerve barrier to engulf relevant axons and myelin-derived debris [21]. Simultaneously, SCs proliferate to form Büngner bands that induce growth cones to engulf damaged proximal axons, and SCs then wrap around the regenerating axons to form myelin sheaths (Figure 1) [9]. The effects of PNI can be compensated by the regeneration of new shoots from lateral branches of undamaged axons and the regrowth of damaged axons [18]. One mechanism involves the regeneration of new shoots from lateral branches of undamaged axons. When PNI occurs, surrounding healthy neurons can sprout collateral branches that extend toward the denervated area. These new extensions can reinnervate the target tissue and partially restore function [22]. Regrowth of the damaged axons themselves is the other mechanism. Severed axons have the capability to regenerate from their proximal stumps. Growth cones form at the severed ends and, guided by various molecular signals and supported by SCs, advance along the original route until they reach their intended targets, and re-establish neural connections [23]. However, experimental evidence suggests that these two mechanisms are insufficient for restoring function in damaged nerves [24].

PNI with a nerve gap requires invasive surgical adjuvant therapy, including end-to-end sutures, grafts, and nerve guide conduits. When the defect exceeds a critical size, autologous nerve grafting is considered the “gold standard” for clinical repair [25]. However, the application of autologous transplantation is limited due to loss of function at the donor site and potential nerve mismatch reactions [26]. NGCs have been developed as a promising alternative for PNI repair and have undergone significant structural and functional adjustments during their application and continued development. Hollow NGCs were the first clinically approved alternative to autologous transplantation to bridge a nerve gap [27]. Although hollow NGCs fulfill the basic requirements for nerve regeneration, including limiting fibroblast infiltration, reducing neuroma formation and scarring, providing mechanical support by connecting injury sites, and facilitating the localized accumulation of neurotrophic factors, their repair efficacy still falls short of that achieved by autologous nerve transplantation [28,29].

**Figure 2:**
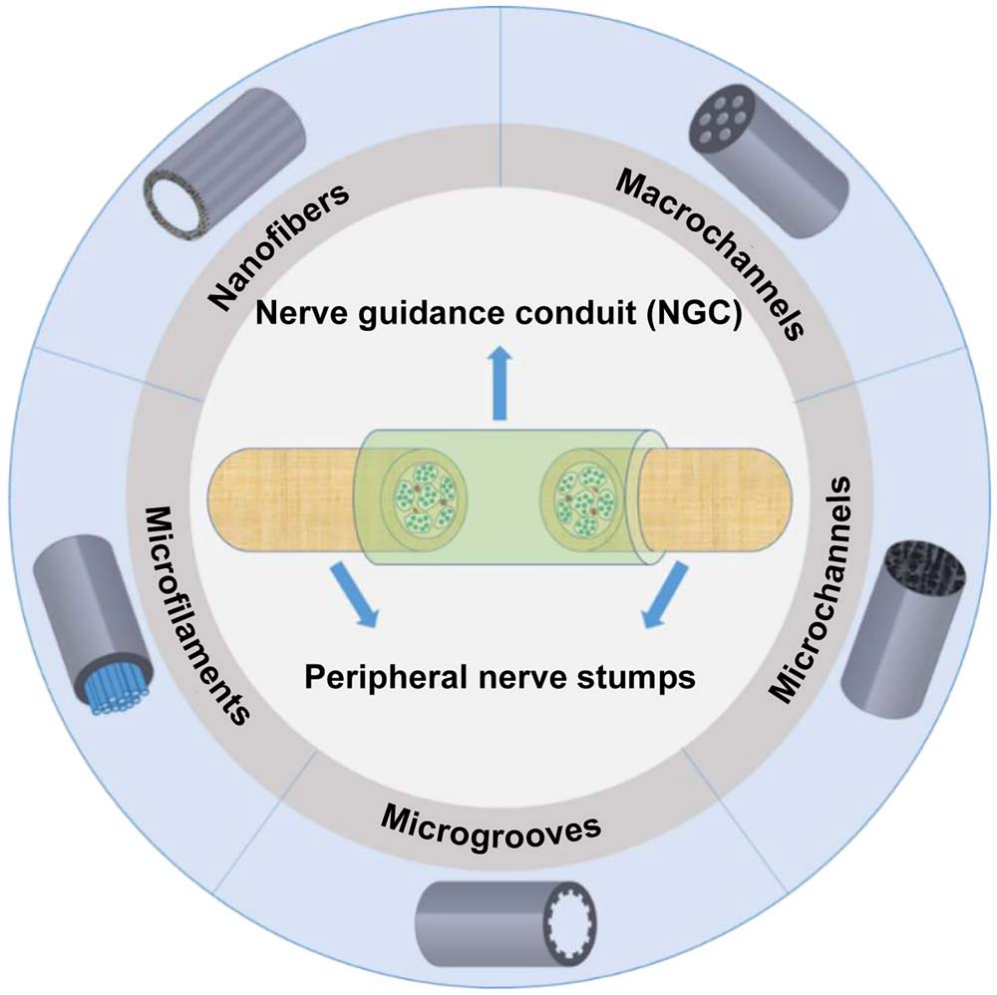
Topology design of NGCs. Reproduced with permission [33]. Copyright 2021, royal. Society of Chemistry

Hollow NGCs are typically functionalized to enhance their clinical applications by being developed into macro-multichannel, microgroove, and filled nerve conduits that provide physical guidance cues for axon growth in the lumen [30–32]. The topological design of NGCs is shown in Figure 2 [33]. Macro-multichannel conduits, fabricated through injection molding, electrospinning, and phase separation, exhibit significant permeability [30,34,35]. This feature facilitates nutrient exchange and promotes dense neuronal cell growth both on the surface and within scaffold channels, providing clear guidance for axonal extension and assisting in the recovery of nerve function [36]. Microgrooves, formed on biomaterial surfaces by microlithography, can significantly influence cellular behaviors such as morphology, alignment, proliferation, migration, and differentiation [37]. Research indicates that microgroove structures significantly improve cellular responses to scaffolds, thereby enhancing the efficacy of nerve repair [38].

In addition, filled nerve conduits focus on internal matrix design to mimic the fascicular structure of peripheral nerves and support cell attachment, migration, and proliferation. These conduits contain fibers, gels, or sponges as internal matrices. Fiber-filled conduits consist of bundled micron-sized strands that provide a greater surface area for protein or neurotrophic factor loading than simple tubular nerve guidance conduits (NGCs), promoting nutrient penetration and nerve repair [39]. Gel-based matrices provide a soft, biocompatible environment that supports cell viability while enabling the incorporation of growth factors or bioactive molecules to further stimulate regeneration [40]. Sponge structures, characterized by their permeability and mechanical support, facilitate 3D cellular interactions and efficient nutrient and waste exchange [41,42]. In summary, multi-channel conduits emphasize the importance of external structural guidance, while filled conduits emphasize the supportive role of the internal microenvironment. Multi-channel and filled nerve conduits offer unique designs and technological advantages that address critical aspects of nerve regeneration.

Furthermore, functional NGCs are grafted with polypeptides, loaded with neural cells and growth factors, and imbued with electrical conductivity, thereby providing biochemical cues to construct a microenvironment suitable for neural regeneration [43–47]. Advances in NGC design, from simple hollow conduits to sophisticated multi-channel and filled conduits, have significantly improved their efficacy by providing physical guidance and supportive microenvironments that mimic natural nerve architecture [48]. Incorporating biochemical and electrical cues into NGCs further enhances neural regeneration potential, underscoring the multidisciplinary progress toward more effective PNI repair strategies. Nevertheless, ongoing research is essential to optimize these technologies and achieve clinical outcomes comparable to autologous grafts. Recently, NGCs in response to electrical signals have received considerable attention.

### Effects of ES on cellular responses and signaling pathways

**Figure 3:**
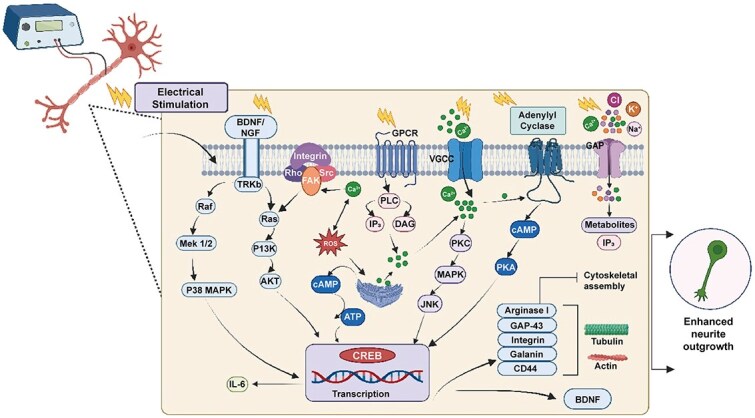
Intracellular signaling pathways associated with electrical stimulation regulate nerve regeneration. Reproduced with permission [57]. Copyright 2025, Elsevier

It is known that nerve cells possess electrical properties. During nerve signal transmission, the cell membrane potential shifts from a resting state to an action potential, rapidly facilitating depolarization and repolarization, returning to the resting state, and completing electrical activity [49]. After PNI, the generation of action potentials is impaired, leading to blocked transmission of electrical signals. ES activates intrinsic cellular mechanisms for nerve regeneration by mimicking natural calcium influx waves. Therefore, ES can stimulate the generation of electrical signals that propagate retrogradely to cells, similar to naturally occurring action potentials, rejuvenating the injury site [50]. ES activates signaling pathways like mitogen-activated protein kinase (MAPK), which controls molecular pathways of specific messenger RNA (mRNAs), such as p38 MAPK, extracellular signal-regulated kinase (ERK), and phosphatidylinositol-3 kinase (PI3K), promoting neurite growth in neural cells (Figure 3).

Specifically, several interrelated calcium ion-dependent signaling pathways within neurons are involved in this process. ES directly affects cellular ion dynamics by facilitating Na^+^ influx and K^+^ efflux while elevating intracellular Ca^2+^ levels through plasma membrane ion channels. Simultaneously, ES induces activation of phospholipase C (PLC) by the G protein–coupled receptor (GPCR), leading to the synthesis of inositol 1,4,5-trisphosphate (IP3) and diacylglycerol (DAG), which subsequently triggers Ca^2+^ release from the endoplasmic reticulum [51]. Subsequently, intracellular calcium ion waves can mediate many downstream effects of ES. Intracellular Ca^2+^ activates PKC and MAPK signaling cascades, triggering JNK and p38 MAPK pathways, which further support cellular responses like cytokine regulation and cytoskeleton assembly. Furthermore, increased levels of Ca^2+^ in neuronal soma induce upregulation of brain-derived neurotrophic factor (BDNF) and its receptor tropomyosin receptor kinase B (TRKb) [52]. Activation of TRKb initiates Ras-MAPK (Raf-MEK1/2-ERK) and PI3K/AKT signaling pathways, promoting the activity of the transcription factor cAMP response element binding protein (CREB), which regulates the expression of genes critical for axon growth and cytoskeletal reorganization, including arginase I, GAP-43, integrins, and CD44. Additionally, intracellular Ca^2+^ can bind to GPCRs on cell membranes, activating adenylyl cyclase and increasing cAMP concentration [53]. The surge in cAMP activates protein kinase A (PKA), whose subunits enter the nucleus and stimulate CREB, which further promotes transcription of regeneration-associated proteins [6,54]. In addition to neurons, the effect of ES extends to SCs and PC12 cells, and SCs activated by ES secrete various neurotrophic factors and express their receptors, including glial cell–derived neurotrophic factor (GDNF), brain-derived neurotrophic factor (BDNF), and its receptor TRKb, thereby promoting axon regeneration and elongation [55,56]. These neurotrophic factors enhance gene expression of various regeneration-associated proteins, including tubulin, galectin-1, actin, growth-associated protein-43 (GAP-43), and neurotrophin-4/5 (NT-4/5) [57]. Collectively, these pathways coordinate cellular responses to enhance axon guidance and nerve regeneration.

ES regulates the nerve cells mentioned above and controls macrophage activity to create an inflammatory microenvironment conducive to neural regeneration. Studies have shown that ES induces intracellular calcium influx by regulating the expression of transient receptor potential (TRP) ion channels in macrophages, activates PI3K and ERK signaling pathways, and enhances phagocytosis efficiency [58,59]. ES influences the behavior of immune cells, particularly macrophage polarization. In a recent study, the combination of NGCs and ES synergistically inhibited the release of inflammatory factors and transformed macrophages from an inflammatory M1 phenotype to a tissue regenerative M2 phenotype, modulating the inflammatory microenvironment and supporting peripheral nerve regeneration [60]. Collectively, ES integrates multiple cellular mechanisms, creating a synergistic environment that supports comprehensive nerve recovery. However, the precise molecular interactions between ES-regulated macrophages and nerve cells warrant further investigation to entirely elucidate the underlying mechanisms driving this therapeutic potential.

### Electroactive biomaterials

**Figure 4:**
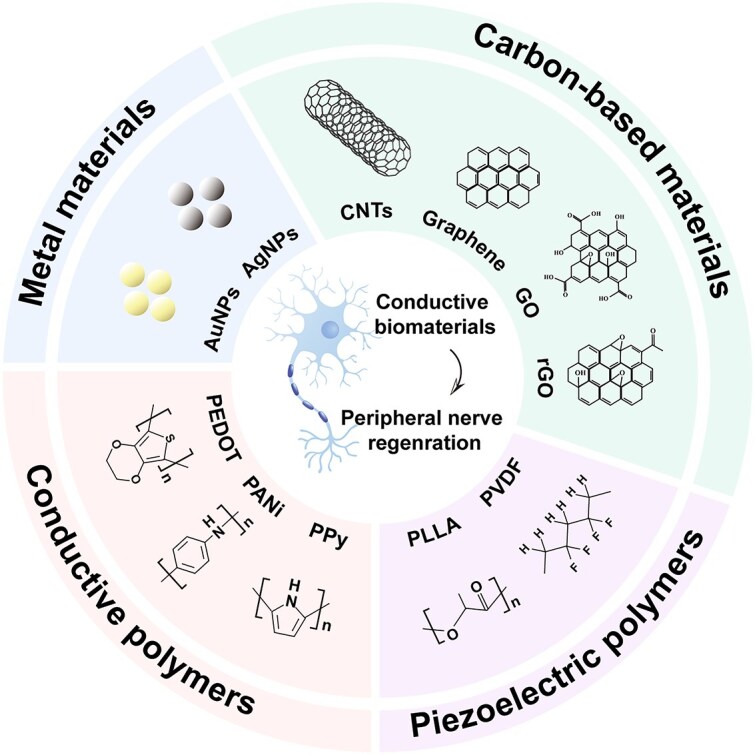
Several crucial electroactive biomaterials are utilized in NGCs

Electroactive biomaterials, such as metals, carbon-based materials, conductive polymers, and piezoelectric materials, have been widely used in PNI to engineer functional NGCs that contribute to the active state of ES (Figure 4). The following section outlines prospective electroactive biomaterials for the fabrication of conductive NGCs. Table 1 lists the characteristics of various electroactive biomaterials.

**Table 1:** Characteristics of various electroactive biomaterials

Electroactive biomaterials	Advantages	Disadvantages	Ref
AuNPs	Excellent conductivity, stability, and corrosion resistance.	High cost, potential toxicity.	[112]
AgNPs	High conductivity and antibacterial properties.	Cytotoxicity, high cost, and poor long-term stability.	[113,114]
CNTs	High conductivity, high mechanical strength, and stability.	Potential toxicity and difficult dispersion.	[115,116]
Graphene	High electrical properties, lower toxicity, and high mechanical properties.	Difficult modification and dispersion, inadequate bioactivity.	[117–119]
GO	Good dispersion and biocompatibility.	Inferior conductivity and mechanical properties.	[120]
rGO	Higher conductivity, thermal stability, and biocompatibility.	Easy agglomeration, potential toxicity.	[121]
PPy	High conductivity, easy synthesis, low cost, and biocompatibility.	Degradation and dissolution challenges.	[122,123]
PANi	High conductivity, stability, and low cost.	Difficulty in degradation and brittleness.	[124]
PEDOT	Low impedance, high conductivity, weak or little toxicity, and high flexibility.	Insoluble in most solvents and complex synthesis.	[125]
PLLA	Self-electrical stimulation, biocompatibility, and degradability.	Acid products, limited mechanical strength.	[126]
PVDF	Wireless ES, stability, processability, and sensitivity.	Poor degradability and low cytocompatibility.	[127,128]

#### Metal materials

Metal materials such as gold nanocomposites and silver nanoparticles have been utilized to repair peripheral nerve regeneration due to their significant electrical conductivity and processability.

#### Gold nanoparticles

Gold nanoparticles (AuNPs) possess favorable electronic properties in which the behavior of electrons is not limited by the energy of singlet atoms but can move freely in a continuous energy range and efficiently transmit electric current [61]. The practical application of AuNPs in PNI is commonly integrated with conductive polymers, rather than relying solely on AuNPs to improve the conductivity of materials. Such composites contribute to improving the overall performance of the material. For instance, a polydopamine-coated gold/polycaprolactone nanoscaffold was developed to increase microvascularization and the number of myelinated fibers, showing promising potential for peripheral nerve restoration [62]. AuNPs have also been incorporated with polyaniline to fabricate conductive reinforced composites through self-assembly. These hybrid composites exhibited higher conductivity than individual materials and induced the differentiation of stem cells into neuron-like cells when exposed to ES [63]. However, the high cost remains a limiting factor for the expansive application of AuNPs.

##### Silver nanoparticles

Silver nanoparticles (AgNPs) are effective in peripheral nerve repair primarily due to their outstanding antibacterial properties and electrical conductivity [64]. Numerous studies have established a correlation between the antibacterial capabilities of AgNPs and neuronal regeneration [65]. Accumulation of reactive oxygen species (ROS) during the initial phase of peripheral nerve injury contributes to inflammation, dysfunctional SCs, and aggravated damage [66]. Therefore, the ROS-responsive process stands as a trailblazer in the realm of peripheral nerve regeneration. The antimicrobial mechanism of AgNPs involves the generation of oxidative stress through uncleared ROS. Recently, Guan *et al*. developed a multifunctional conductive hydrogel by incorporating polydopamine-modified AgNPs, cellulose nanocrystals/polypyrrole (CNC/PPy), and polyvinyl alcohol (PVA) into a composite material. This hydrogel exhibited desirable conductivity and effective nerve repair in combination with ES [67]. Additionally, AgNPs scaffold may promote laminin adsorption rate, myelin sheath thickness, nerve conduction velocity, and potential amplitude of damaged sciatic nerves [68]. The antibacterial and neuron-promoting effects of AgNPs make them a promising nanomaterial.

#### Carbon-based materials

Previous studies of carbon-based materials used in NGCs have primarily concentrated on carbon nanotubes (CNTs) and graphene-based materials due to their exceptional electrical conductivity and ease of modification.

##### Carbon nanotubes

**Figure 5:**
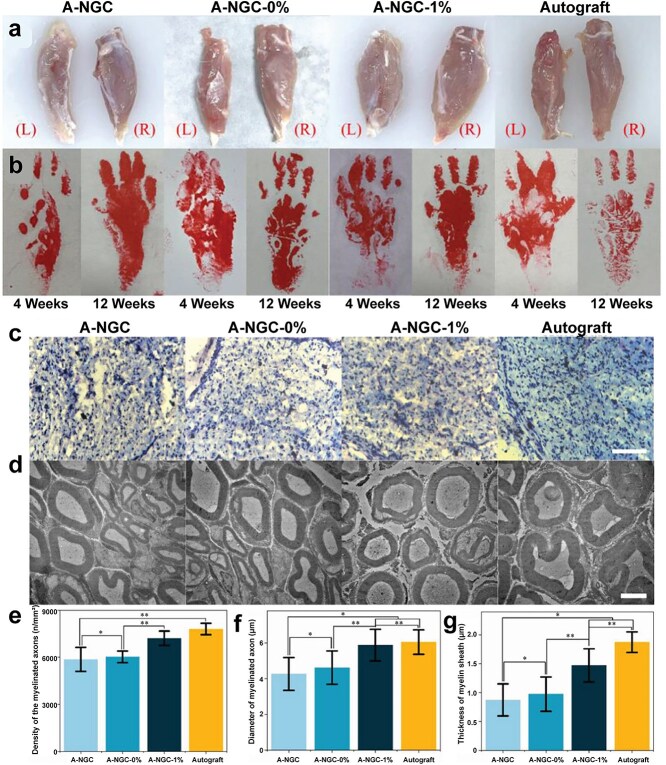
Evaluation of gastrocnemius muscle recovery and nerve regeneration at 12 weeks postoperatively. (a) Photographs of separated gastrocnemius muscles. (b) Photographs of footprints. (c) TB staining of the cross-section of regenerated nerves. (d) TEM images for regenerated nerves. (e) Density and (f) diameter of myelinated axon. (g) Thickness myelin sheath. Reproduced with permission [73]. Copyright 2024, Wiley

CNTs, which consist of multiple layers of carbon atoms, can be categorized as single-walled CNTs (SWCNTs) and multi-walled CNTs (MWCNTs). CNTs are renowned for their excellent electrical, mechanical, and thermal conductivity properties and have been extensively used in biomedical scaffolds over the past decade [69,70]. Previous research has indicated that CNTs can enhance neuronal adhesion, growth, cell differentiation, and intracellular signaling circuits [71]. In particular, the combination of CNTs with ES promoted these processes and further enhanced neural repair outcomes. Incorporating CNTs into degradable chitosan (CS) composite fibers has been found to improve the strength and conductivity of the fibers without causing inflammation *in vivo* [72]. Sun *et al*. proposed NGCs containing 1% MWCNTs, which demonstrated comparable results to autografts in restoring muscle and nerve function (Figure 5) [73]. However, the hydrophobic property of CNTs may limit their application to nerve regeneration [74]. Moreover, unmodified CNTs have been found to exhibit certain cytotoxicity [49].

Among NGCs, Huang *et al*. prepared a laminin-modified CNTs/CS neural conduit, which improved electrical conductivity and the cell adhesion rate, showing potential to guide nerve axon regrowth [75]. The effect of nerve regeneration was further enhanced by optimizing the electrical properties of CNTs to make them more responsive to ES. Besides, oxidation-processed CNTs promoted biocompatibility and nerve growth while suppressing foreign body reactions [76]. Carboxylated MWCNTs significantly facilitated PC12 cell differentiation by upregulating the expression of the neurotrophin signaling pathway–associated TrkA/p75 receptors and Pincher, Gap43, and TH proteins, implying the potential application of carboxylated MWCNTs in neurodegenerative diseases [77]. These studies approved that the unique physicochemical and biocompatibility characteristics of CNTs were the underlying basis for their positive applications.

##### Graphene-based materials

Graphene-based materials exhibit distinct properties for nerve regeneration, including superior electrical conductivity, specific surface area, significant mechanical strength, and biocompatibility [78]. Graphene-based materials have attracted considerable attention because they can preserve electrical transmission between proximal and distal nerve stumps, especially when combined with ES, facilitating the nerve repair process more efficiently [79]. Among graphene-based materials, graphene, graphene oxide (GO), and reduced GO (rGO) are widely used in neural tissue regeneration. Qian *et al*. integrated cross-linked graphene-laden poly(e-caprolactone) (PCL) porous NGCs with polydopamine (PDA)/arginyl glycyl aspartic acid (RGD) to enhance neural activity, improve axon regeneration, and promote myelin repair [80].

Although graphene exhibits excellent electrical properties, its application to peripheral nerve regeneration is limited by issues related to surface modification, combination with other materials, and dispersion in solution [81]. Negatively charged carboxylate compounds on GO can enhance colloidal strength and hydrophilicity, making it more suitable for surface attachment, growth, and differentiation of nerve cells. Compared to graphene, GO is more widely used in tissue engineering due to its simple properties and ease of processing [82]. When coated on Antheraea pernyi silk fibroin (ApF)/polylactic acid-co-caprolactone (PLCL) scaffolds, GO could enhance the biological behaviors of SCs, induce differentiation of PC12 cells, and successfully repair a 10 mm sciatic nerve defect [83].

However, electrical conductivity and mechanical properties are reduced by the presence of oxygen-containing functional groups in GO [81]. The rGO, a graphene derivative, serves as an intermediate structure between graphene and GO with fewer oxygen-containing functional groups and improved electrical properties. Song *et al*. recently illustrated that the incorporation of rGO into the poly (lactide-co-trimethylene carbonate)/gelatin nanofibers considerably improved the electrical conductivity of the matrix and dramatically enhanced nerve restoration, particularly under ES [84]. The rGO is frequently used instead of graphene in nerve tissue engineering due to its increased accessibility, superior electrical conductivity, and reduced cost.

#### Conductive polymers

Conductive polymers (CPs), as novel organic materials, can regulate biological activities with or without ES [85]. Polypyrrole (PPy), polyaniline (PANi), and poly (3,4-ethylene dioxythiophene) (PEDOT) are the most extensively studied CPs in nerve regeneration.

##### Polypyrrole

PPy is a carbon–carbon double and single-bond conjugated polymer with electrical conductivity, environmental stability, and biocompatibility [85]. The use of PPy as a coating for neural conduits is one of the most effective and convenient ways to enhance the conductivity of biomaterials. PPy-coated electrospun PLCL scaffolds with ES showed superior cell migration, neurite outgrowth, axon diameter, myelin thickness, and functional recovery of sciatic nerve defect compared to nonstimulating conductive conduits [86,87].

**Figure 6:**
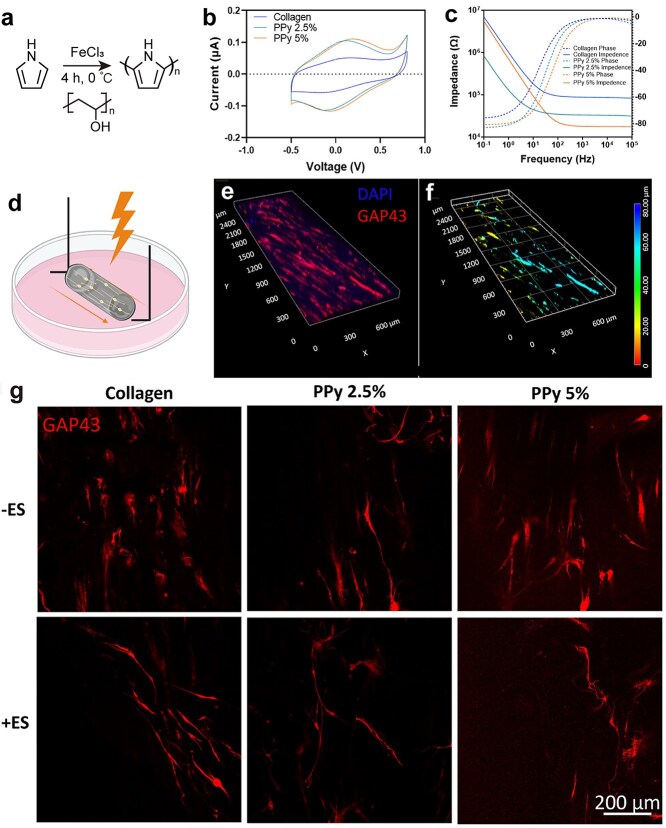
Electronic characterization and neurite extension of bioelectronic constructs. (a) Scheme of the polypyrrole (PPy) synthesis. (b) Cyclic voltammetry and (c) electrochemical impedance spectroscopy of the constructs. (d) Schematic diagram of nerve cell regulation by electrical stimulation. (e) Confocal micrographs of neurite extension. (f) Confocal micrographs of neurite extension after 72 h. (g) Single slices of confocal immunofluorescence micrographs with immunostaining of GAP-43 in neurons within the constructs. Reproduced with permission [90]. Copyright 2024, Wiley

However, the undesirable hydrophobicity of PPy interferes with cell–protein interactions, impeding nerve repair. To ameliorate this unsatisfactory property, researchers have developed composite biomaterials with improved characteristics [88,89]. A 3D bioelectronic conductive scaffold consisting of conductive PPy nanoparticles embedded in an aligned collagen hydrogel. The PPy-loaded construct supported a 1.8-fold increase in the neurite length of primary rat dorsal root ganglion (DRG)–derived primary rat neurons when ES was applied, showing significant nerve regeneration potential (Figure 6) [90]. The charge transfer of ions in PPy allows it to switch between oxidized and reduced states. In the conductive form, PPy is positively charged and can bind to negatively charged molecules, such as proteins and drugs [91]. An electrospun PLCL/SF with NGF-loaded conductive tannic acid (TA)–PPy-L-arginine, glycine, and L-aspartate (RGD) hydrogel was fabricated, which could activate the PI3K/AKT signaling pathway in PC12 cells and significantly improve axon thickness of myelin sheath, angiogenesis, and motor function of sciatic nerve defect [92]. The integration of ES and conductive PPy NGCs appeared to facilitate functional sciatic nerve recovery, emerging as a potential solution for repairing long-distance nerve deficits.

##### Polyaniline

**Figure 7:**
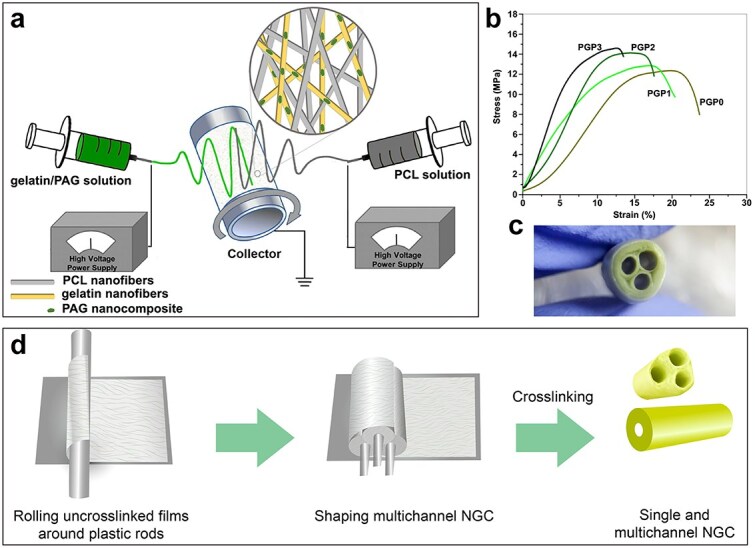
A multi-channel conduit fabricated by poly(e-caprolactone) and gelatin, incorporated with polyaniline/graphene. (a) Illustration of the electrospinning process of poly(e-caprolactone) (PCL) and gelatin incorporated with polyaniline/graphene bilayer membranes. (b) Stress–strain curves of conduits. (c) Photo of the multi-channel conduit. (d) Illustration of the fabricating process of conduits. Reproduced with permission [94]. Copyright 2020, Wiley

Polyaniline (PANi) is a widely used traditional CP owing to its high electrical conductivity, easy synthesis, and thermal stability. However, the π-conjugated bonds in the structure of PANi cause deficiencies like brittleness and poor solubility, which leads to chronic inflammation due to wear and debris formation during its long-term presence *in vivo* [93]. Blends with natural or synthetic biopolymers can efficiently overcome these limitations. For instance, PCL and gelatin were incorporated with PANi/graphene (PAG) nanocomposites to conduct electricity in the form of double-electrospun membranes formed into a multi-channel conduit. Then, this double-layer conduit exhibited excellent conductivity of 10.8 ± 0.6 × 10^−5^ S/cm and fair tensile strength of 3.52 ± 1.3 MPa (Figure 7) [94]. Borah *et al*. constructed a conductive PANi nanofiber–dispersed CS nanocomposite scaffold using a one-step surface functionalization approach that improved viability, adhesion, and diffusion of primary adipose-derived mesenchymal stem cells (MSCs) [93]. Yi *et al*. synthesized a conductive hydrogel by PANi on carboxymethyl CS (CMCS), which had good electrical conductivity matching the sciatic nerve and increased nerve conduction velocity, enhanced expression of neuronal axon-specific proteins, and induced axon extension and remyelination of the sciatic nerve [95].

##### Poly (3,4-ethylene dioxythiophene)

PEDOT as a polythiophene derivative is commonly utilized in tissue engineering because of its conjugated bonds and structural characteristics exhibiting interaction with neural cells [96]. In addition to its superior electrical conductivity compared to PPy, PEDOT is considered biocompatible and can be excreted by the kidney [97]. Although PEDOT has limited solubility and hydrophilicity, PSS is a highly dispersed polymeric dispersant that can react with PEDOT to form the water-soluble, stable, and dispersed PEDOT:poly (styrenesulfonic acid) (PSS) polymer [98,99]. Babaie *et al*. fabricated an electrospun conductive polyvinyl alcohol (PVA)/PEDOT:PSS scaffold that improved the differentiation of rat MSCs by upregulating β-tubulin, nestin, and enolase. When combined with ES, this scaffold further enhanced cellular response and nerve repair by mimicking the properties of natural nerve tissue [100]. A recent artificial peripheral nerve conduit made of polyvinylidene fluoride (PVDF)/PLCL/PEDOT was designed to integrate piezoelectric properties and electrical conductivity to facilitate the reconstruction of nerve conduction and motor function while activating the PI3K/AKT-Nrf2 signaling pathway to establish an appropriate anti-inflammatory, immune microenvironment [101]. It is conceivable that PEDOT may serve as an efficacious conductive polymer to promote peripheral nerve regeneration.

#### Piezoelectric polymers

Piezoelectric materials are capable of generating electrical pulses by converting mechanical stress without external energy sources [102]. Piezoelectric polymers with biocompatibility are more commonly used in nerve tissue engineering than other piezoelectric materials.

##### Poly-L-lactic acid

**Figure 8:**
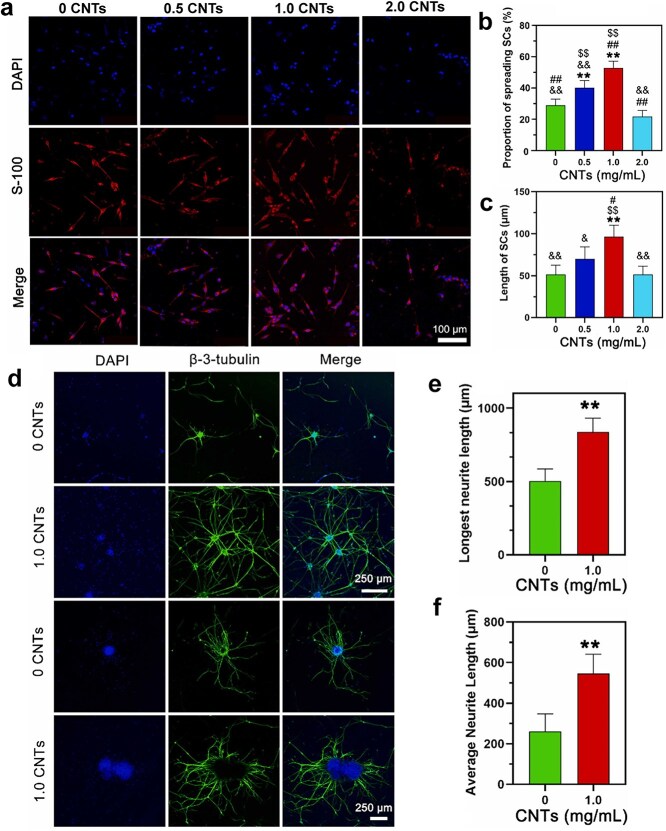
Biocompatibility and neurite development of DRG neurons on carbon nanotubes@gelatin methacryloyl/poly(L-lactic acid) scaffold. (**a**) S-100 fluorescence staining at 7 days. (**b**) Spreading proportion and (**c**) average length of SCs cultured on scaffolds. (**d**) Representative fluorescence images cultured on scaffolds. (**e**) Quantitative analysis of maximum neurite lengths. (**f**) Quantitative analysis of average neurite lengths. Reproduced with permission [106]. Copyright 2023, Elsevier

**Figure 9:**
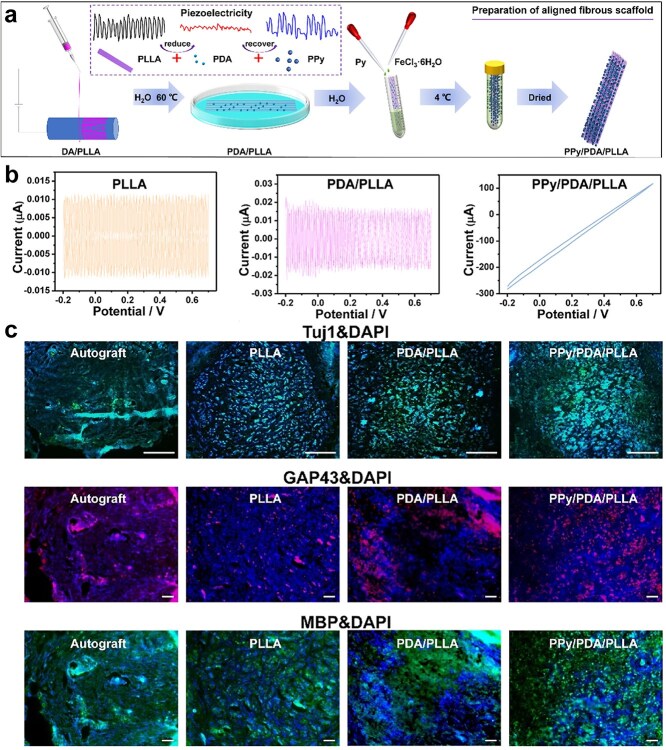
Fabrication and characterization of polypyrrole/polydopamine (PDA)/poly(L-lactic acid) electrospun scaffolds. (**a**) Illustration of the preparation of the aligned scaffold. (**b**) Cyclic voltammograms of samples. (**c**) Tuj1, GAP43, and MBP immunofluorescence stainings were performed on nerve transection after 12 weeks. Reproduced with permission [107]. Copyright 2023, Wiley

Poly(L-lactic acid) (PLLA) is an ideal piezoelectric polymer known for its exceptional biodegradability and biocompatibility, which has been used in nerve regeneration [103]. The study of electrospun PLLA nanofibers demonstrated that changing the orientation of C=O dipoles of PLLA can induce piezoelectricity [102]. However, certain limitations have been identified in relation to the practical application of PLLA scaffolds. It is difficult for neural cells to adhere and grow on the hydrophobic surface of PLLA scaffolds. Furthermore, the decomposition of PLLA produces an acidic microenvironment, which is not optimal for nerve repair [104]. These shortcomings of PLLA can be solved by mixing with other compounds, providing novel solutions for nerve regeneration [105]. For example, a soft, self-powered, and electroconductive CNT-blended gelatin methacryloyl (GelMA)/PLLA scaffold was fabricated, significantly improving the adhesion of SCs and axonal outgrowth of DRG. Meanwhile, the scaffold facilitated the recovery of axon outgrowth and motor function at 12 weeks postimplantation (Figure 8) [106]. In another study, a conductive PPy/polydopamine (PDA)/PLLA electrospun scaffold was constructed to improve the hydrophilicity and cellular compatibility of PLLA, which was beneficial for the stimulation of differentiation of SCs and the extension and alignment of DRG neurons. This hybrid scaffold activated calcium and AMP-activated protein kinase signaling pathways, effectively bridging the 10 mm sciatic nerve deficit (Figure 9) [107]. These findings indicated that the piezoelectric properties of PLLA enabled the nerve conduit to generate electrical impulses without requiring an external power source. When combined with other conductive materials, this setup synergistically established an electrical signal transmission pathway during nerve defects, effectively guiding nerve regeneration by directing cellular electrical signals.

##### Polyvinylidene fluoride–based materials

**Figure 10:**
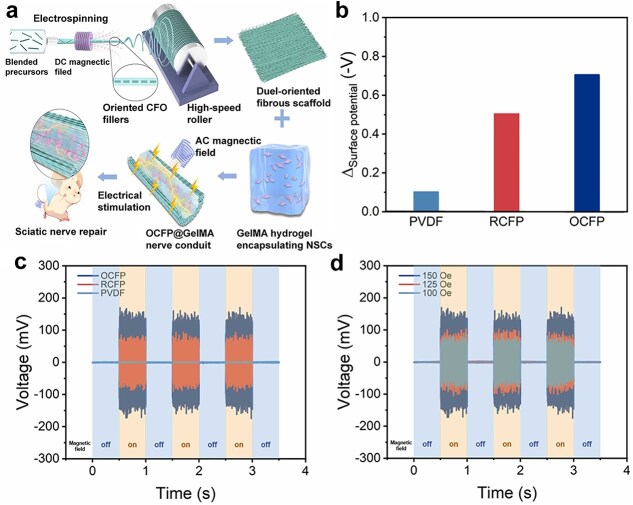
Fabrication and *in situ* magnetoelectric coupling properties of oriented fibrous filler cobalt ferrite (CoFe_2_O_4)_ and oriented polyvinylidene fluoride nanofibers (OCFP), random fibrous filler CoFe_2_O_4_ (RCFP), and polyvinylidene fluoride (PVDF) scaffolds. (**a**) Schematic of the fabrication of the novel wireless charging magnetoelectric scaffold. (**b**) Potential difference statistics for different scaffolds. (**c**) OCFP, RCFP, and PVDF fibrous scaffolds under open circuit output voltage under 150 Oe alternating current magnetic field. (**d**) OCFP fibrous scaffolds under 150, 125, and 100 Oe AC magnetic field. Reproduced with permission [111]. Copyright 2024, Elsevier

After subtle mechanical deformation, PVDF-based materials can produce transitory surface charges and exhibit piezoresponse due to the dominant β-crystalline polar phase, which is a potential biopolymer for facilitating electrically stimulated neuronal regeneration [108]. Pi *et al*. designed electrospun piezoelectric nanotubes composed of PCL and PVDF, which significantly promoted the proliferation of SCs and neuronal cells, and restored complex motor functions and axonal maturation in a 15 mm sciatic nerve defect [109]. PVDF was utilized to develop an electrospun SF/PVDF-co-hexafluoropropylene/Ti3C2Tx (MXene) composite conduit, which increased the output voltage to 100 mV of piezoelectric properties, induced proliferation of SCs, and promoted axonal myelination [110]. Further research on the piezoelectric properties of PVDF showed that the aligned PVDF nanofibers combined with the aligned cobalt ferrite (CoFe_2_O_4_, CFO) fibrous fillers improved the β-phase content and provided stable and controllable wireless ES for PNI repair (Figure 10) [111]. Under these findings on the piezoelectric effect of peripheral nerves, PVDF-based materials indicate a safe and feasible method for long-distance neural defects and provide the foundation for the design of smart NGCs.

### Current clinical use and surgical indications of electroactive biomaterials

Utilizing advanced materials in clinical practice for peripheral nerve repair, particularly within conductive NGCs, requires a thorough understanding of material science and medical application. The transition of the listed electroactive biomaterials from experimental research to clinical practice has been selective and cautious. Only a subset of these materials has found its way into clinical applications. Polymers like PLLA are widely used due to their biocompatibility and ability to be tailored into various forms suitable for tissue engineering [129,130]. PLLA is Food and Drug Administration (FDA)-approved and utilized in resorbable sutures and scaffolds, indicating its safety and efficacy in surgical settings [131]. Similarly, while not all polymers have direct clinical applications as NGCs, some, like PEDOT:PSS, show promise in enhancing electrical conductivity in experimental models but are still primarily in the preclinical stage [132].

Surgical indications for using electroactive biomaterials in NGCs would primarily include cases where traditional treatments fail to provide satisfactory outcomes [133]. For metallic nanoparticles like AuNPs and AgNPs, and nanomaterials like CNTs, graphene, GO, and rGO, their integration into clinical practice remains under investigation [134–136]. Although these materials exhibit exceptional properties that promote neural regeneration, concerns about long-term biocompatibility and potential toxicity must be thoroughly addressed before clinical approval can be considered. The same applies to conducting polymers like PPy, PANi, and PEDOT, which show great promise, but further research is required to ensure safety and effectiveness *in vivo* [137].

### Fabrication methods of conductive nerve guidance channels based on electroactive biomaterials

**Figure 11:**
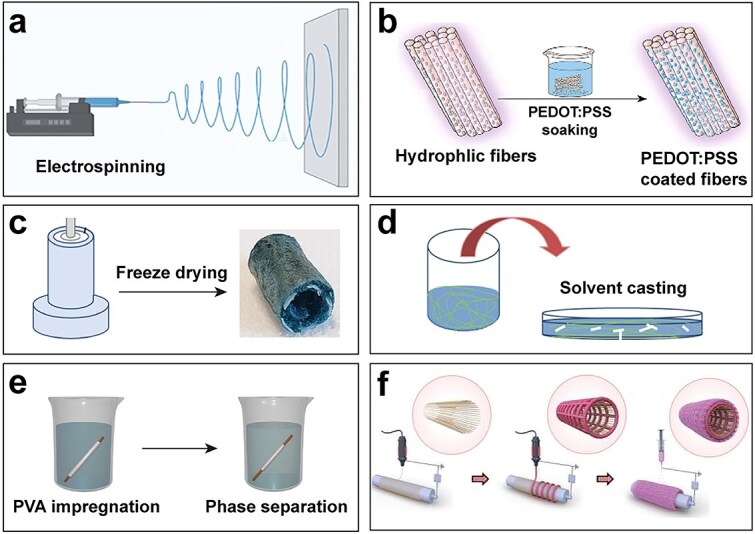
The fabrication methods of conductive nerve guidance conduits. (**a**) Electrospinning. Reproduced with permission [159]. Copyright 2024, Wiley. (**b**) Dip-coating. Reproduced with permission [142]. Copyright 2024, Elsevier. (**c**) Freeze drying. Reproduced with permission [160]. Copyright 2023, Multidisciplinary Digital Publishing Institute. (**d**) Solvent casting. Reproduced with permission [161]. Copyright 2017, De Gruyter. (**e**) Phase separation. Reproduced with permission [162]. Copyright 2023, Elsevier. (**f**) 3D printing. Reproduced with permission [156]. Copyright 2023, Wiley

Due to the limited solubility and processing challenges associated with most electroactive biomaterials, various fabrication techniques have been developed to produce NGCs to fulfill the specific preparation requirements of these conduits. A series of fabrication methods of conductive NGCs based on electroactive biomaterials are primarily summed up, including electrospinning, dip-coating/salt-leaching technique, freeze drying process, centrifugal casting, phase separation, and 3D bioprinting (Figure 11). These preparation strategies are detailed in the following sections.

#### Electrospinning

Electrospinning is a direct spinning method driven by high-voltage electrostatic forces for producing random or aligned nanofibers with diameters ranging from nanometers to micrometers that closely resemble the structure of the extracellular matrix to guide cellular alignment and mediate rapid intercellular communication in nerves [138]. Single-wall carbon nanotubes were dispersed in the spinning solution by mixing, and conductive aligned polycaprolactone/gelatin/single-walled carbon nanotube nerve conduits were prepared by electrostatic spinning, which exhibited good electrical conductivity and supported the growth and proliferation of nerve cells [139]. However, several issues of electrospinning still deserve attention, such as poor reproducibility, incomplete evaporation of toxic solvents, and difficulties in customization. Additionally, the uneven dispersion of most electroactive biomaterials in the electrospun solution can compromise the electrical conductivity and mechanical properties of the nerve conduit [140]. Therefore, many studies have combined electrospinning and dip-coating techniques.

#### Dip-coating

A uniform conductive layer can be rapidly formed on the surface of existing matrix NGCs by dip-coating, and the conductive polymer can be chemically polymerized *in situ* on the surface of the matrix material [141]. For instance, Liu *et al*. developed porous polylactic acid–glycolic acid (PLGA)-coated PEDOT:PSS conductive nerve conduits that exhibited superior electrical conductivity and mechanical properties, promoting neural cell growth without cytotoxicity. When combined with ES, these conduits significantly enhanced the recovery of hindlimb function in rats and improved therapeutic outcomes for nerve regeneration [142]. In another study, PEDOT:PSS solution was coated on PCL electrospun fiber mats to improve the conductivity of the material [143]. However, there are problems with dip-coating, such as difficulty in precisely controlling the coating thickness, which will affect the mechanical properties of the substrate material, and long-term stability depends on the bonding force between the coating and the substrate [144,145].

#### Freeze drying

The freeze-drying process facilitates the preparation of porous structures, which generates interconnected porous structures by sublimation of the solvent after freezing [146]. A gelatin/alginate/graphene composite polymer scaffold prepared by freeze-drying technology exhibited electrical conductivity and biodegradability, supported PC12 cell attachment, and mimicked the ECM of natural neural tissue, thereby maintaining structural integrity and enhancing cell permeation and nutrient diffusion [147]. There are some limitations to the application of this technology, such as time-consuming, the requirement for specialized equipment, and high cost [148].

#### Solvent casting

Solvent casting has the advantages of easy operation, low cost, and is suitable for mass production, and the ability to adjust the physical properties of mixed materials according to the material ratio [149]. The PVDF/PEDOT:PSS/ionic liquid membranes prepared by solvent casting have good electrical conductivity and can be adjusted as needed, potentially valuable for applications in neural tissue engineering [150]. However, the lack of topographic guidance and insufficient porosity in solvent-cast conduits limit the wider application of this method [151].

#### Phase separation

Phase separation is a common technique for fabricating polymeric scaffolds in which the target polymer is dissolved in a mixture of immiscible solvents. Upon heating, a saturated solution separates into polymer-rich and polymer-poor phases. The subsequent rapid cooling, or quenching, triggers phase separation, with the polymer-poor phase being eliminated through processes such as evaporation or extraction. In contrast, the polymer-rich phase solidifies into a porous structure [152]. Thermotropic phase separation was used to fabricate a scaffold of PCL and carbon nanofibers, followed by electrospinning to produce PCL/collagen nanofiber sheets. These components were subsequently assembled by putting the nanofiber sheets into the cavity of the scaffold, forming an electrically conductive nerve conduit [153]. However, the materials that can be used with phase separation are limited [154].

#### 3D printing

3D printing stands at the forefront of manufacturing technologies for NGCs, facilitating the precise realization of complex scaffold designs required for advanced neural tissue engineering applications [155]. Conductive multiscale filled conduits with electrospun PCL/collagen nanofibers as the sheath, 3D-printed reduced graphene oxide/PCL microfibers as the scaffold, and 3D-printed PCL microfibers as the internal support have been developed for peripheral nerve regeneration. These conduits exhibit excellent permeability, mechanical stability, and electrical conductivity, significantly enhancing SCs’ elongation and growth and promoting neurite outgrowth of PC12 cells, thereby facilitating effective peripheral nerve regeneration [156]. Digital light processing (DLP) printing was used to fabricate complex hydrogel structures using GelMA and CS, while PEDOT nanoparticles were introduced by interfacial polymerization to create conductive pathways within hydrogel structures. In *in vivo* experiments in rats, nerve conduits made of 3D-printed conductive hydrogels effectively promoted nerve regeneration and facilitated muscle recovery [157]. However, 3D printing faces the challenges of limited print resolution, poor solubility, and fragile conductive materials [158].

In conclusion, each fabrication method for conductive NGCs based on electroactive biomaterials possesses distinct advantages and limitations, catering to specific application domains. Advances in technology continue to refine these methods, enhancing their ability to address increasingly precise and diverse biomedical requirements. Moreover, there is a growing trend toward integrating multiple fabrication techniques to develop multifunctional conductive NGCs, thus better supporting the complex demands of peripheral nerve regeneration. This integrative approach underscores the evolving sophistication of tissue engineering strategies aimed at functional recovery and repair in neurological tissues.

### Clinic progress and challenges of conductive nerve guidance channels based on electroactive biomaterials and electrical stimulation

The pathophysiological process of PNI is very complex, and as mentioned earlier, only a few NGC products have been approved by the FDA. Currently, conductive NGCs based on electroactive biomaterials are still in the research and development stage. Conductive NGCs based on electroactive biomaterials represent a promising approach in peripheral nerve repair, leveraging the intrinsic electrical properties of neural tissue. These materials can mimic the natural environment of neurons, providing a scaffold that supports axonal regeneration while delivering electrical stimuli to enhance neurite outgrowth. Recent clinical progress has demonstrated the feasibility and potential benefits of conductive NGCs [163,164]. Studies have shown that electroactive polymers such as polypyrrole (PPy), poly(3,4-ethylenedioxythiophene) (PEDOT), and polyaniline (PANI) can be incorporated into NGCs to facilitate nerve regeneration [57]. In particular, PEDOT:PSS electrocorticogram electrodes have been tested in 30 human subjects [132]. Most of the research on conductive materials in peripheral nerve regeneration is in the preclinical stage, while ES has been widely studied and applied in the clinical treatment of peripheral nerves. A recent clinical trial assessed the safety and efficacy of an electrical vagus nerve stimulation (VNS) neurostimulator in patients with multidrug-refractory rheumatoid arthritis. The device significantly improved patients’ disease activity scores over a 12-week period through short bursts of ES multiple times per day using specific parameters (10 Hz frequency and 250-ms pulses) compared to sham stimulation (device implanted but not activated) [165]. Furthermore, in a clinical trial of VNS for the rehabilitation of patients with arm injury following ischemic stroke, 108 patients were implanted with a VNS device by investigators and randomly assigned to receive either true VNS treatment or a sham stimulation group. After the addition of VNS (using 0.8 mA, 100 ms, 30 Hz stimulation pulses lasting 0.5 s each) to standard rehabilitation treatment for 6 weeks, significant improvement in upper extremity function was demonstrated compared to the control group (Fugl–Meyer assessment). A 90-day follow-up showed that a clinically significant response was maintained by 47% of patients in the VNS group, compared to 24% in the control group [166].

Despite encouraging advancements, several challenges remain in translating conductive NGCs from scientific research to clinical application. A major hurdle is achieving long-term stability of the electroactive components within the body. Degradation or loss of conductivity over time could undermine therapeutic efficacy [167]. Additionally, optimizing electrical properties to match physiological conditions without causing adverse effects is crucial yet complex. Integration of conductive elements must also consider biocompatibility and tissue integration, ensuring minimal inflammatory response and robust mechanical strength [168].

Furthermore, standardization of fabrication techniques and quality control measures are required to ensure reproducibility across different batches of NGCs. Regulatory approval processes pose another challenge, requiring extensive preclinical data demonstrating safety and efficacy before clinical application can be pursued. In summary, while conductive NGCs based on electroactive biomaterials present an exciting avenue for enhancing peripheral nerve repair, addressing the associated technical and regulatory challenges will be critical to their successful implementation in clinical practice. Continued research and development are essential to refine these technologies and bring them closer to routine clinical use.

### Innovative studies for peripheral nerve injury treatment

Previous studies have demonstrated that ES is predominantly utilized in laboratory or clinical settings using standard electrical stimulators. These devices conventionally rely on external alternating current or direct current power sources, which are often costly and lack portability [57]. Furthermore, in clinical applications, the necessity of using predetermined wires to connect the power supply unit to the target tissue introduces several challenges. This wired approach is associated with an increased risk of tissue infections, complications, secondary surgical injuries, and other adverse effects for patients [169]. In contrast, wireless ES offers significant advantages by enhancing both portability and safety [170,171]. Recently, the use of wireless ES for nerve repair has garnered considerable attention from researchers, highlighting its potential as a promising alternative in this field. In neuroscience engineering, specific and noninvasive magnetic and optogenetic stimulation present broad application prospects as promising alternatives to ES [172–174].

The capability of magnetic fields to remotely control magnetically responsive materials demonstrates significant clinical translational potential due to their excellent tissue penetration and ease of manipulation [175]. In the field of tissue engineering, magnetoelectric materials have emerged as a novel approach for delivering wireless ES. These materials can convert magnetic energy into electrical energy through a coupling mechanism involving magnetism, force, and electricity when exposed to an external magnetic field [176]. By combining magnetic scaffolds with an applied magnetic field, a synergistic effect can be achieved, enhancing functional recovery after tissue repair. This approach reduces side effects while improving treatment effectiveness and flexibility [177]. It has been reported that a chitosan@artemisia sphaerocephala conduit containing polydopamine (PDA)-modified Fe_3_O_4_ nanoparticles to achieve non-invasive magnetic stimulation, which promotes repair and functional recovery of PNI [178]. Liu *et al*. developed a conductive conduit incorporating graphene nanocoatings and Fe_3_O_4_ nanoparticles, combined with wireless ES that generates microcurrents through alternating magnetic fields. This conduit promoted SCs proliferation, migration, and intercellular communication while accelerating neuronal axon extension. *In vivo* studies demonstrated that the conduit significantly enhanced motor function recovery and neural tissue growth, achieving outcomes comparable to the autograft method [179]. The potential of combining ES with magnetic stimulation for future clinical applications was confirmed through experimental validation, underscoring its promising prospects. However, current research on the precise mechanisms of the beneficial effects, safety, and efficacy of magnetic stimulation is rather limited, and further exploration in this area will help optimize its clinical application and expand its therapeutic potential.

Optogenetics is a technique that selectively utilizes light to manipulate specific functional types of neurons. ES activates all types of nerve cells without distinction, whereas optogenetics can selectively stimulate distinct cells at different stages [45]. Application of photostimulation to nerves with specific protein expression can trigger selective neuromodulation to avoid potential off-target effects that may occur during ES [180]. Many studies have shown that the application of optogenetics to PNI may promote axonal growth and nerve regeneration by facilitating the activation of neuronal cells and secretion of associated nerve growth factors [181,182]. Optogenetics has promising applications, but photostimulation requires much higher energy for neural activation compared to ES [183]. Additionally, when employing ES independently, a certain current intensity is necessary to activate neurons, which may inadvertently lead to damage to surrounding tissues. In contrast, the combination of light and ES can utilize low-intensity light to presensitize modified neurons, and low-intensity ES that would be inadequate for neuronal activation becomes sufficient to trigger neural activity [184]. Matarazzo *et al*. investigated that the combined action of optogenetics and ES lowered the ES threshold of the sciatic nerve and enhanced specific nerve responses [185]. Although optogenetics has shown promising applications in PNI, there are still some limitations for application, such as heat generated during light irradiation that may lead to other side effects, the complexity of constructing specific protein expression vectors in response to stimulation, and the persistence of protein expression [186,187].

## Conclusions

In summary, the molecular mechanism of ES regulation on nerve cells, the primary electroactive biomaterials, and the synergistic effect are systematically introduced in nerve tissue engineering. Peripheral nerve regeneration involves complex interactions of multiple cells and intricate biological responses. ES and electroactive biomaterials can significantly influence nerve regeneration independently and collaboratively. Many challenges remain for the production of clinically approved NGCs, including the standardized parameters of ES, the underlying regeneration mechanism of electroactive biomaterials, the toxicity of degradation products, and the effects of long-term *in vivo* presence, all of which affect the practical application of NGCs. Furthermore, the development of NGCs may combine these technologies, such as electrical and optogenetic stimulation and electrical and magnetic stimulation, which will improve repair outcomes for long nerve gap repair. However, further research is needed to optimize these findings and fully elucidate their mechanisms to translate research findings into clinical treatments. This review aims to improve the understanding of the synergistic effects of ES and electroactive biomaterials in the peripheral nerve and provide inspiration for the continuous improvement of functional NGCs.
